# Performance Evaluation Strategies for Eye Gaze Estimation Systems with Quantitative Metrics and Visualizations

**DOI:** 10.3390/s18093151

**Published:** 2018-09-18

**Authors:** Anuradha Kar, Peter Corcoran

**Affiliations:** Department of Electrical & Electronic Engineering, National University of Ireland, Galway H91 TK33, Ireland; peter.corcoran@nuigalway.ie

**Keywords:** eye gaze estimation, eye tracker, performance evaluation, metrics, visualizations, mobile devices, graphical user interface, open source, standardization

## Abstract

An eye tracker’s accuracy and system behavior play critical roles in determining the reliability and usability of eye gaze data obtained from them. However, in contemporary eye gaze research, there exists a lot of ambiguity in the definitions of gaze estimation accuracy parameters and lack of well-defined methods for evaluating the performance of eye tracking systems. In this paper, a set of fully defined evaluation metrics are therefore developed and presented for complete performance characterization of generic commercial eye trackers, when they operate under varying conditions on desktop or mobile platforms. In addition, some useful visualization methods are implemented, which will help in studying the performance and data quality of eye trackers irrespective of their design principles and application areas. Also the concept of a graphical user interface software named GazeVisual v1.1 is proposed that would integrate all these methods and enable general users to effortlessly access the described metrics, generate visualizations and extract valuable information from their own gaze datasets. We intend to present these tools as open resources in future to the eye gaze research community for use and further advancement, as a contribution towards standardization of gaze research outputs and analysis.

## 1. Introduction

**Figure 1:**
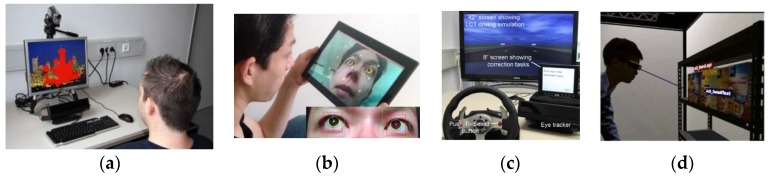
Eye gaze applications in various consumer platforms (from left: (**a**) desktop [2], (**b**) tablet [3], (**c**) automotive [4], (**d**) head-mounted [5] eye tracking setups).

Research works on eye gaze estimation typically present their results in a wide range of ways. While the commonly used measure of gaze tracking accuracy is angular resolution (in degrees), other metrics such as gaze recognition rates (in percentage) and shifts between estimated and target gaze locations (in pixels or mms) are used frequently. These metrics are not interrelated and sometimes not clearly defined, which leads to ambiguities in evaluating and comparing performances of gaze tracking systems. Table 1 below shows the statistics derived from a recent literature review done by the authors [1] surveying nearly 200 research articles on gaze based algorithms and applications, which highlights the current diversity in metrics used for representing gaze estimation performance. It may be observed from the table that although use of angular resolution as a metric is common, the uncorrelated metrics like percentage recognition rates and pixel distances are also used in many works. Column 2 of the table shows the total number of papers surveyed for each consumer platform or eye gaze use-case category. The other columns present the different metrics, with the number in each cell of them representing the number of papers where each type of metric is used. Figure 1 below shows illustrations of eye gaze applications in different consumer platforms like desktop, mobile devices, automotive and head mounted systems. 

Another sparsely investigated issue in gaze research is about studying the impacts of different error sources (i.e., system and user variables) on gaze tracking performance. It was highlighted in [1] that gaze estimation systems on various user platforms face varied operating conditions, which are not well described in conventional research or product literature. Such factors which possibly influence a gaze estimation system’s accuracy include user distance, display properties of the screen where gaze is tracked and user head pose variations. Additionally, for eye tracking on mobile platforms like handheld devices, platform motion and orientation may greatly alter the claimed accuracy of an eye tracker during real life operations. At present, research works commonly do not report impact of these factors or present their results in any uniform graphical format. 

### 1.1. Problem Statement

As described above, there are definite requirements of methods, firstly for detailed analysis of eye tracking data to learn about the tracker system characteristics under variable operating conditions and secondly for attaining homogeneous and fully defined gaze accuracy metrics. Therefore in this work, for comprehensive evaluation of generic eye trackers, a set of metrics and visualization methods are derived by analyzing the data collected from an eye tracker. To develop these metrics and visual tools, experiments with the eye tracker are performed with a group of subjects on two different user platforms (desktop and tablet). The gaze experiments are done on the desktop and tablet devices under controlled variation of tracking conditions such as changes in user distance, user head-pose and platform orientations to study their impacts on gaze tracking accuracy of the tracker. Using the gaze data collected from experiments, several metrics are derived which not only specify tracking accuracy but also look for specific trends in the gaze data that indicate ways to achieve best tracking performance from the tracker. Visualization tools are then built by aggregating the experimental data to understand and compare tracking performance under these varying circumstances. Finally, all the described metrics and visual methods are built into a graphical user interface software with which users can readily explore their own collected gaze datasets, study their tracker characteristics and save the results without delving into details of the source code. 

### 1.2. Purpose, Scope and Structure of the Paper

The purpose of this paper is to introduce some metrics and visualizations that may be helpful to determine the data quality from generic eye trackers when they operate under variable operating conditions. For this, a series of experiments are done to collect data from a commercial eye tracker and its operating conditions were varied, to build specific metrics and visuals that reflect these conditions. Two platforms were used, a desktop and a tablet mounted with the eye tracker to collect gaze data using the same stimuli and data logging process. The tablet was used to especially study the impact of platform orientation variations which is unique to this platform. The metrics defined here were applied on the collected data and the results are used to demonstrate how these metrics may be used by any eye tracker. Similarly, the visualizations are meant to show how these may be used in any gaze tracking experiment to visually inspect gaze data quality and aggregate test results. 

At this point it is also important to outline the scope of this work. It may be noted that in this work we do not intend to evaluate a particular eye tracker. We neither want to classify the used tracker or its data as good or bad or determine their suitability for any application. In other words, here we are not judging an eye tracker itself, but are only using eye tracking data to implement our proposed metrics and visuals. We note that eye tracker data patterns change with varying operating conditions and we expect the developed metrics to represent these changes quantitatively and the visualizations to show this graphically. Aspects like why the tracker behaved in a certain way under certain operating conditions, or analyzing if the accuracy levels are acceptable, are out of scope for this work. This is because analyzing the underlying reasons for data variability is a dedicated body of research, but comprises of a different direction of work with respect to the topic presented here.

As for the structure and organization of the paper contents, there are two major sections here, Section 4 on the Metrics and Section 5 on the Visualizations. Section 4 has several Section 4.1, Section 4.2, Section 4.3 and Section 4.4 and sub-subsections such as Section 4.1.1, Section 4.1.2, Section 4.2.1 and Section 4.2.2, Section 4.3.1, Section 4.3.2, Section 4.3.3 and Section 4.3.4. Each of these sub-subsections present a gaze data evaluation metric and corresponding results from implementing the metric on gaze data collected from relevant experiments. For each metric description, the content is split into “Method” which describes the concept of the metric and “Example results” which displays the outcome from testing the metric on collected data in tabular or graphical form. Similarly, in Section 5 the contents of Section 5.1.1, Section 5.1.2, Section 5.1.3, Section 5.1.4 and Section 5.1.5, Section 5.2.1, Section 5.2.2, Section 5.2.3 and Section 5.2.4 are split into “Method” and “Example results”. A summary of related research is provided in Section 2, the experimental details in Section 3 and conclusion to this work is in Section 6. 

## 2. Related Works: Accuracy Estimates and Visualizations in Gaze Research

In this section, the status of accuracy metrics and data visualizations used in contemporary eye gaze research works is reviewed in Section 2.1 and Section 2.2, and the contributions of this work in this direction are highlighted in Section 2.3.

### 2.1. Previous Work on Evaluation of Gaze Estimation Systems

Eye gaze research has progressed to include multiple user platforms with increasing number of applications in human computer interactions [6,7,8] marketing, psychology and ecommerce [9,10]. A major aim of eye tracking system design is to achieve high accuracy and consistency in results. A wide range of eye tracking systems and algorithms have been developed over the past few decades which have been reviewed in [11,12]. Dedicated research works on evaluation and reporting of gaze data quality and standardization of has been presented in [13,14,15,16]. More works such as [17,18,19] discuss on the systematic performance evaluation of eye tracking systems including frameworks, databases and virtual environments. 

Reference [13] discusses the lack of common standard measures in for eye data quality which affect eye tracker usage and research on eye movements. It provides a detailed description of what is meant by eye data quality and how data accuracy and precision affects measurements of eye movement features such as dwell time, number and duration of fixations and pupil size and further describes standardized means of reporting data quality. Reference [14] describes the use of an artificial eye to implement methods for objective evaluation of eye tracking data quality. First, the temporal accuracy and latency of an eye tracker is tested using an artificial saccade generator. Then an artificial pupil is mounted on a computer controlled moving platform to provide biologically similar eye movements. Reference [15] studies the impact of eye data collection conditions on the factors such as calibration, pupil identification, fixation detection and gaze analysis. They implement mobile eye tracking in an outdoor environment and report that all stages of processing eye tracking data must be tailored to the data collection conditions. Reference [16] discusses the need for development of a standard application programming interface API for eye trackers in order to build applications using gaze data. The aim is to have a unified way to interact with eye-tracking systems and receive gaze data using same protocol regardless of the eye tracker.

Works such as [20,21,22,23,24] present results from the evaluation of specific commercial eye trackers from companies such as Eyetribe, SMI, Tobii and GazePoint. Reference [20] compares the accuracy, precision and sampling rates of the Eye Tribe (The Eye Tribe ApS, Copenhagen, Denmark) and SMI RED eye trackers (SensoMotoric Instruments, Teltow, Germany) and shows the impact of system setups such as user sitting position, height of stimulus screen, height of eye tracker, user tracker distance and frame rate on the data from the trackers. Reference [21] also discusses the evaluation and comparison of the EyeTribe and SMI RED eye trackers by concurrently recording data from them and measuring parameters like accuracy, data loss and fixation counts for application in cartographic research. Reference [22] presents the comparison of several eye trackers such as Eye Tribe, Tobii EyeX (Tobii Technology Inc., Danderyd, Sweden), Seeing Machines faceLAB (Seeing Machines, Fyshwick, Australia), Smart Eye Pro and Smart Eye Aurora (Smart Eye AB, Gothenburg, Sweden) to study features such as gaze tracking accuracy, precision, impact of glasses and data loss. Reference [23] discusses the objective evaluation of the Gazepoint GP3 eye tracker (Gazepoint Research Inc., Vancouver, BC, Canada), and studies its capabilities with respect to pupil dilation metrics under cognitive loads and luminance conditions, whereas [24] evaluates the Tobii EyeX tracker for accuracy, precision and latency parameters to determine its suitability for behavioral studies. 

However, from these works, it may be observed that apart from accuracy, precision and latency, no other evaluation metrics for eye tracker evaluation have been studied in detail. Also within these works there remains inhomogeneity in the definition of performance metrics as some of them report gaze accuracy in degrees while some in pixel measures. These aspects have been discussed in our previous paper [1] which describes the existing inhomogeneity in gaze data accuracy measures and the need for development of more intricate gaze tracking performance metrics. It also proposed the concept of a dedicated evaluation framework for all round performance assessment of eye trackers. 

### 2.2. Data Visualizations in Gaze Research 

In [25] a comprehensive overview and classification of gaze data visualization methods is presented. The methods are grouped on the basis of visualization type (e.g., statistical/spatio-temporal, 2D vs. 3D visualizations), eye tracking data type (fixation, scanpath, smooth pursuit, saccades) and stimuli (point-based for studying gaze distribution and areas of interest (AOI)-based for understanding AOI interrelationships). Other classifications include: static (image based) and dynamic (video based), active and passive stimulus based visualizations. Conventionally gaze data is aggregated using heat maps and fixation maps, on which several improvements have been proposed, such as in [26] (modifying transparency of the heat map depending on gaze data) and [27] (real-time heatmap generation and visualization of 3D gaze data). In [28], gaze visualization with parallelized rendering on a GPU (graphics processing unit) platform is proposed. The aim is to speedup heatmap creation on video stimulus, while [29] proposes dynamic heatmap visualizations coupled with user visual focus information on different backgrounds (dark, blurred, fog). Several novel visual approaches have in been reported in [30,31,32]. In [30,31] gaze stripes and color bands are described in which a sequence of gaze point images are aligned in a timeline to analyze and compare the viewing behavior of multiple participants in a clutter-free manner. Kurzhals et al in [32] presents a visual analytics method to interpret eye movement data recorded for dynamic stimuli such as video or animation. Scanpath is an useful gaze data visualization method which is used in [33] to differentiate scanning behavior of participants over stimulus images. It is a very detailed work on studying viewing patterns of users looking at natural scene images by applying scanpath comparison metrics like string edit distance, sample based, linear distance etc. on eye tracking data. Other innovative gaze data representations can be found in [34,35] and a popular open source gaze data analyzer is described in [36].

The above literature survey shows that a wide range of state-of-the-art methods for gaze data analysis and techniques for visualization are currently present in gaze research. However, it can be seen that these methods are mostly directed towards exploration of eye movement characteristics (such as speed, direction and duration), understanding its relation to human behavior [37] such as attention, cognitive load assessment, regions and sequence of interests [38] or studying visual saliency [39]. No visualization work is found which is dedicated towards evaluation of eye trackers themselves, or studying the data characteristics of eye trackers under variable operating conditions. 

### 2.3. Requirement of Well-Defined Accuracy Metrics and Performance Visualization Tools in Eye Gaze Research: Contributions in This Work

It is observed that currently there is a lack of well-defined metrics for comprehensive evaluation of eye trackers and standard open source visualization methods for understanding their performance characteristics are also not available. Keeping these in mind, this paper puts forward some effective solutions for current and future gaze researchers for complete characterization of their eye tracking devices. The contribution in this paper includes the development and description of a set of accuracy metrics and visualization methods, and a software user interface for accessing these to produce and save results, with all of these being meant for use on data from any generic eye/gaze tracker to completely specify its performance. 

Benefits of the metrics presented in this paper include: (a) derivable simply from eye gaze spatial coordinates and corresponding ground truth data (b) provide quantitative measures of an eye tracker’s performance and therefore can be used to compare multiple eye tracking systems and algorithms (c) can help to estimate impact of operating conditions on eye tracking data (d) can be adapted to different computing systems, display sizes and resolutions (e) may be used irrespective of eye tracking algorithm and hardware (f) reveal specific trends in gaze data which indicate ways to improve tracking performance. 

With the proposed visualization methods, one may: (a) have a quick look at an eye tracker’s data characteristics without going deeper into the tracking system/algorithm (b) visually compare tracking performance and data quality of multiple eye trackers (c) present volumes of experimental data in single or few figures. With the graphical user interface named GazeVisual developed in this work, which is described in more detail in later sections of the paper, a generic user of any gaze based application may implement all the metrics and visualization functions described in this work on their gaze data without going into details of source code. Also, owing to the open source nature of the software, it may be adapted by eye gaze researchers to suit their individual research purposes, while advanced programmers may also contribute towards its functional extensions.

## 3. Experimental Methodology

### 3.1. Experimental Setup and Workflow

**Figure 2:**
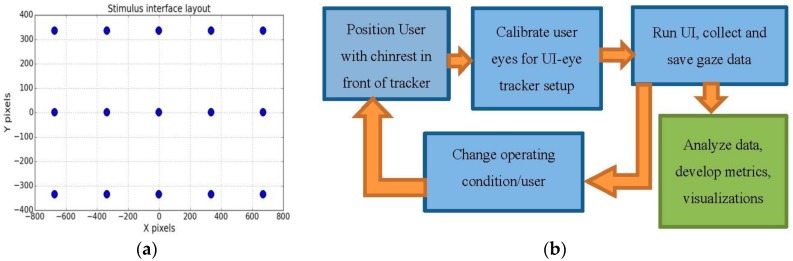
(**a**) Shows the layout of the stimulus interface (UI) with AOIs (circles) where a user has to look during data collection. (**b**) Shows the experiment flowchart for data collection.

Eye tracking experiments are performed on desktop and tablet platforms for gaze data collection. The setup details are provided in Table 2 and comprises of a remote eye-tracker mounted on the screen of a desktop or a tablet device, which display a visual stimulus interface (UI). In the UI, a moving dot sequentially traces a grid of (5 × 3) locations over the display screen as shown in Figure 2a (it shows the static view of the screen locations traced by the dot). The UI dot radius is 10 pixels and it stops at each location for 3 s before moving on to the next. The angular extent of the UI stimulus grid is 30 degrees of visual angle at 45 cm distance. The UI is synchronized with the eye tracker to collect gaze data during an experimental session. The locations traced by the dot are henceforth called areas of interest or AOIs whose on-screen positions are known in pixel coordinates. The collected data comprises of a participant’s gaze coordinates on the display and corresponding time stamps as estimated by the tracker, while AOI locations form the ground truth. All data are stored in comma separated values (CSV) files.

Experiments are done by positioning users in front of a computer screen mounted with the tracker while their head is fixed with a chin rest. During the experiments, the UI is run on the desktop or tablet screen and users are asked to follow the moving dot as it moves. This ensures that the user’s fixation distance is closest to stimuli locations. The eye tracker calibration uses 9 points and the calibration stimulus comprises of dots appearing at the corners, top and bottom locations of the display. The AOI stimulus dot size is comparable to the eye tracker calibration stimulus dot. After the calibration procedure, the calibration quality is validated using a validation procedure provided by the eye tracker software, and for poor calibration, the process is repeated.

A typical experimental workflow is shown in Figure 2b. The procedure followed during one complete experimental session is presented in the top three blocks. After completion of each session, certain system or user parameter is changed, such as registering a new user, varying user distance or head pose, and then the experimental sessions are repeated with the new condition. Outcome of the experiments is the collected gaze dataset (block on bottom right corner) which is then analyzed and used for development of metrics and visualizations for performance evaluation of the eye tracker.

### 3.2. Setup Coordinate System and Eye Movements

**Figure 3:**
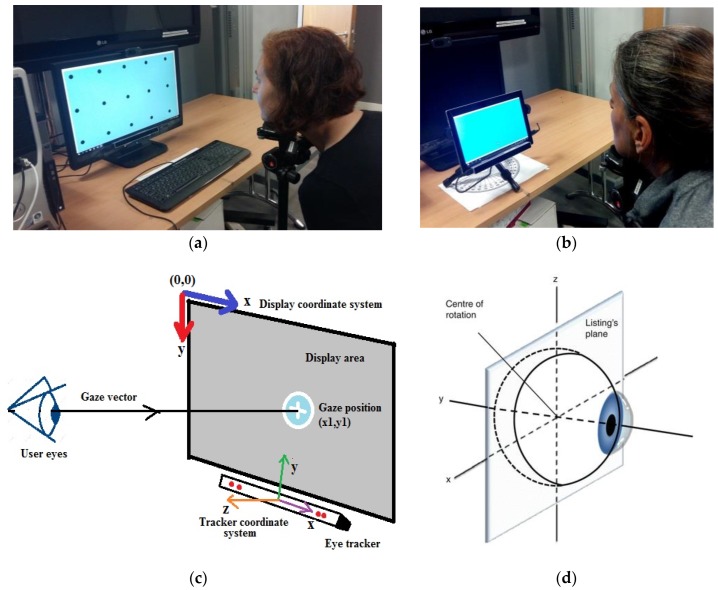
The figure on top left (**a**) shows the eye tracker under test mounted on a desktop computer display along with a participant seated in front of it (desktop screen shows the AOI layout in static form). (**b**) Shows a tablet mounted with the eye tracker using the same UI and experimental workflow as that used for the desktop. (**c**) Shows the display and eye tracker coordinate systems for this work and (**d**) shows the eye movement coordinate systems with Listing’s plane and Fick’s axes.

Figure 3a,b show the experimental environment, while Figure 3c explains the setup coordinate system in which gaze data is collected during experiments. The display screen is the area used to show the stimulus points from both the UI and tracker calibration routines. The display coordinate system is aligned with the display of the desktop or tablet used in this work and its origin is the upper left corner of the display screen. The eye tracker coordinate system has its origin at the center of the frontal surface of the eye tracker which is aligned with the center of the display screen. The tracker x-axis points horizontally towards the user’s right, the y-axis points vertically towards the user’s up and the z-axis points towards the user, perpendicular to the front surface of the eye tracker. The gaze data comprise of eye locations of a user tracked by the eye tracker and mapped into the 2D coordinates of the display screen. The gaze x, y data of user eye locations using this coordinate system has (0,0) at the display screen center and z data represents the user distance from the tracker starting from 0 at the tracker.

Describing the kinematics of the eye requires the definition of reference positions and coordinate systems. Primary position of the eye is one such reference and defined as the position the eye assumes when the subject is looking straight ahead, while the head is kept upright. Movements of the eye around the primary position may be defined using several coordinate systems such as Fick’s, Helmholtz and Euler [40]. Out of these, in this work, the Fick’s coordinate system is assumed for describing eye movements along with the Listing’s plane (Figure 3d). The axes of Fick have a head-fixed vertical axis and eye-fixed horizontal axis [41]. In the Fick’s axes, the x-axis is the transverse axis passing through the center of the eye at the equator and vertical rotations of the eye occur about it. The y-axis passes through the pupil and torsional rotations occur about this axis. The z-axis is a vertical axis; and horizontal rotations occur about this. Listing’s equatorial plane contains the center of eye rotation and the x and z axes while the y-axis is perpendicular to it. Eye-fixed reference frames as the Fick’s axes [42] are similar to mechanical mounting system like gimbals where one axis is for panning left or right (yaw or horizontal axis) and one for tilting up or down (pitch or vertical axis). Torsional movements of the eye are not considered in this work. 

### 3.3. Eye Tracking Experiments Conducted for the Development of Gaze Accuracy Metrics and Visual Tools

The purpose of the experiments described in this work is to collect eye tracking data under variable operating conditions that may affect an eye tracker working on a desktop or tablet platform. The collected data is used to implement and test evaluation metrics and visualizations and described in Section 4 and Section 5. The following experiments were conducted in this work. (a) User distance variability experiments: In these experiments, gaze data is collected with user-eye tracker distances of 45, 60 and 75 cm. The terminologies “UD45”, “UD60”, “UD75” are used in this paper for referring to experiments/datasets obtained from the tracker at the distances of 45, 60 and 75 cm, respectively. (b) Head-pose variability experiments: This is relevant to studying the effect of a user’s head pose on gaze tracking accuracy of the eye tracker. By head pose, the position of a user’s head in 3D space in terms of roll, pitch and yaw (RPY) angles is meant here. During the experiments, a user is seated at a fixed distance (60 cm) from the tracker and is asked to vary their head position to different rotation angles (head pose in roll, pitch, yaw) while looking at the UI on the display screen and their gaze is tracked on the UI. The head position is also tracked simultaneously using a head pose model that measures head pose angles in RPY values with 1 degree of accuracy. Gaze tracking errors corresponding to different head-poses are then analyzed (c) Platform orientation experiments: Eye tracking on dynamic platforms like tablets face some unique challenges since their positions vary frequently and result in variable orientation of eye trackers which are mounted with the tablet screen. To quantize the impact of tracker orientation on gaze data, experiments are performed in which the orientation of the tablet device mounted with the eye tracker is varied with respect to the user at fixed platform roll, pitch and yaw angles. Eye tracking data is collected for each tablet orientation with the same test UI as used for the desktop system. The objective of these experiments is to study impact of platform orientation variations on eye tracking data characteristics.

## 4. Deriving Evaluation Metrics for Eye Tracking Devices and Algorithms

Eye tracking accuracy is typically measured in terms of the difference between the real stimuli positions and the corresponding measured gaze positions and expressed as angular, pixel or distance measures. However, accuracy expressed in this way provides little information about detailed tracker characteristics and impact of variable operating conditions. Therefore, a set of metrics are derived and presented here, which aim at describing the quality of eye gaze data by taking the characteristics of a gaze tracking system into consideration. The metrics derived in this work are classified into four categories, namely angular accuracy metrics, statistical metrics, sensitivity metrics and a new metric based on Receiver Operating Characteristic (ROC), which are described in the subsections below.

### 4.1. Angular Accuracy Metrics

#### 4.1.1. Gaze Angular Accuracy

Different research groups working in eye gaze most often use independent accuracy metrics or do not describe their accuracy calculation in detail. In order to facilitate the interpretation of a generic eye tracker’s specifications, as well as to provide an objective way to compare different systems, it is important that the same accuracy metrics are used by everyone and each metric be clearly described. That is, eye tracking data must be analyzed in a standard and consistent way. The purpose of this section and its sub-sections is to describe such a common set of calculations, which may be used to measure and compare the accuracy of eye trackers by using only their raw data outputs (and ground truth locations), irrespective of their tracking algorithm or platform. 

***Method:***

Starting from raw gaze x,y pixel coordinates of the left and right eye (X_left_, Y_left_ & X_right_, Y_right_ respectively) obtained from the tracker, the angular accuracy of gaze tracking is derived below [43]:

*Gaze Point Coordinates in Pixels:*

(1) $$\mathrm{GazeX}\;=\;\mathrm{mean}\left(\frac{{X\;}_{\mathrm{left}}{+\;X}_{\mathrm{right}}}{2}\right),\;\mathrm{GazeY}\;=\;\mathrm{mean}\left(\frac{{\;Y}_{\mathrm{left}}+Y_{\mathrm{right}}\;}{2}\right)\;$$

*Gaze Position in mm of on Screen Distance:*

XPos (mm) = µ * GazeX, YPos (mm) = µ * GazeY(2)

where µ is the pixel size of the particular monitor which is calculated depending on the monitor screen dimensions and pixel resolution. The calculation for the factor µ is shown as: µ = d_m_/d_p_ where d_p_ is the screen diagonal size in pixels as obtained from Equation (3) below, w_p_ is the screen width in pixels, h_p_ is the screen height in pixels and d_m_ is the diagonal size in mm (converted from inches):

(3) $$d_{p}=\sqrt{w_{p}^{2}+h_{p}^{2}}$$

For example, when our experiments were performed on a 22 inch (diagonal) monitor operating at 1680 × 1080 pixel resolution, we had d_p_ = 1981, d_m_ = 558.8 and µ = 0.28.

*On Screen Distance (OSD):*

When the origin of the gaze coordinate system is at (x_pixels_, y_pixels_), the on-screen distance of a user’s gaze point is the distance between the origin and a certain gaze point. It is given by Equation (4), with the offset being defined as the distance between the tracker sensor and lower edge of display screen. In our case, the tracker is attached directly below the screens so the offset value is 0 and x_pixels_,y_pixels_ = (0,0), i.e., origin is the center of the screen:

(4) $$\mathrm{OSD}\;(\mathrm{mm})=\;µ\sqrt{\left({\left(\mathrm{GazeX}\;-\frac{x_{\mathrm{pixels}}}{2}\right)}^{2}+{\left(y_{\mathrm{pixels}}\;-\;\mathrm{GazeY}\;+\;\frac{\mathrm{offset}}{\mathrm{pixelsize}}\right)}^{2}\right)}$$

*Gaze Angle Relative to the Eyes:*

Using trigonometry, the gaze angle of a point on screen relative to a user’s eyes is calculated as:

(5) $${\mathrm{Gaze}\;\mathrm{angle}\;(\theta )\;=\;\tan}^{-1}(\mathrm{OSD}/Z)$$

where Z is the distance of the eye from the screen. The distance between the eye and the gaze-point (mentioned as EGP) is estimated from 3D Cartesian geometry as:

(6) $$\mathrm{EGP}\;\left(\mathrm{mm}\right)\;=\sqrt{\left({\left(\mathrm{GazeX}\;\right)}^{2}+{\left(\;\mathrm{GazeY}\right)}^{2}+{\left(Z\right)}^{2}\right)}$$

*Pixel Distance between Ground Truth and Estimated Gaze Point (pix_dist):*

As described in Section 3, the AOI locations (x, y coordinates) displayed on the screen during the experiment form the ground truth for data collections. Using Cartesian geometry, the shift between the ground truth coordinates (GT.X, GT.Y) and tracked gaze locations (GazeX, GazeY) is given by:

(7) $$\mathrm{pix}\_\mathrm{dist}\;(\mathrm{pixels})=\surd \left({\left(\mathrm{GT}.X\;-\;\mathrm{GazeX}\right)}^{2}+{\left(\mathrm{GT}.Y\;-\;\mathrm{GazeY}\right)}^{2}\right)$$

*Angular Accuracy:*

The gaze estimation accuracy (or error as referred in this paper) of an eye tracker is expressed in absolute units (degrees) as the angular deviation between ground truth and estimated gaze locations. Using the estimates of gaze angle, pixel distance and the distance between eye and the gaze point from Equations (5)–(7), the formula for estimating gaze tracking accuracy may be calculated as:

(8) $${\left(µ\;*\;\mathrm{pix}\_\mathrm{dist}\;*\;\cos(\mathrm{mean}(\theta )\right)}^{2})/\mathrm{EGP}$$

***Example Results:***

Using these equations and data from our experiments, the mean angular accuracy of the eye tracker used in this work is found to be between 3 to 5 degrees for a user tracker distance of 45 cm, 2 degrees for a distance of 60 cm and 0.9 to 2 degrees for 75 cm respectively. The results from the above calculations are also used to estimate gaze error throughout this paper to compute other gaze data metrics and implement visualizations.

#### 4.1.2. Gaze Yaw and Pitch Angular Accuracies

The calculations in Section 4.1.1 indicate user gaze angular accuracy by considering a primary eye position. The eye also undergoes rotational motion which leads to different eye orientations relative to the head. The two eye rotation variables are gaze yaw and pitch, where the yaw variation corresponds to left-right and pitch variation corresponds to top-bottom eye movements.

***Method:***

The gaze yaw and pitch angles are derived as follows:

(9) $${\mathrm{Gaze}\;\mathrm{pitch}(\theta \mathrm{pitch})\;=\;\tan}^{-1}{(\mathrm{GazeY}/Z),\;\mathrm{Gaze}\;\mathrm{yaw}\;(\theta \mathrm{yaw})\;=\;\tan}^{-1}(\mathrm{GazeX}/Z)$$

where, GazeX, GazeY and Z are defined above in Equations (1), (2) and (5). To estimate the gaze yaw and pitch errors from the experiments, the ground truth yaw and pitch angles are first calculated using the position of the AOI dots as they appear and move on the screen. The ground truth pitch and yaw value for each AOI dot with screen coordinates (AOIx, AOIy) are given by:

(10) $${\mathrm{AOI}\;\mathrm{pitch}\;=\;\tan}^{-1}{(\mathrm{AOIy}/Z),\;\mathrm{AOI}\;\mathrm{yaw}=\;\tan}^{-1}(\mathrm{AOIx}/Z)$$

***Example Results:***

**Figure 4:**
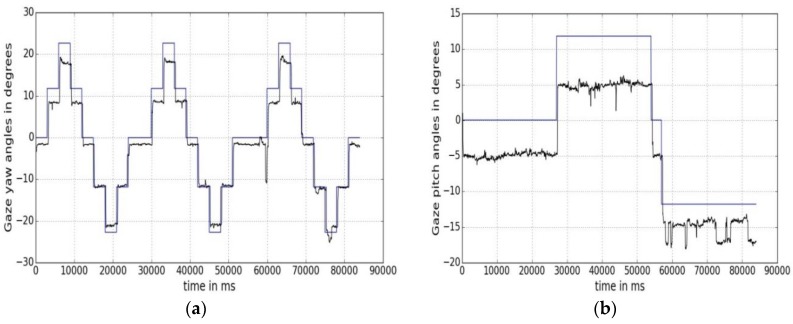
(**a**) On the left shows the gaze yaw angle variations (overlaid with ground truth) with time as recorded for one person during one complete experimental session (i.e., time starts when the user starts gazing at AOI locations appearing on the screen and stops at the last AOI). (**b**) On the right shows the gaze pitch (and ground truth) variations during one session.

Using Equations (9) and (10), the gaze yaw and pitch angle values along with ground truth yaw and pitch values for one user gaze data during one experimental session are plotted against time in Figure 4a,b, respectively. In Figure 4a, the blue line represents the ground truth yaw angles, as calculated from Equation (10). Each step in the curve represents a different AOI position on the screen. The black line shows the variation of a user’s gaze yaw angle with time as estimated by Equation (9). In Figure 4b, ground truth AOI pitch angles are shown in blue and estimated gaze pitch angles in black. 

The maximum and minimum (±) gaze yaw and pitch values for 5 users seated at 45 cm from our tracker were found to be ±22 degrees and ±12 degrees respectively. Gaze pitch errors are seen to be higher in magnitude than gaze yaw error values, and are about 4.5 degrees at 45 cm while yaw error is about 2.6 degrees. Thus, gaze tracking errors are not just scalar values but also have directional components which are not reflected if only mean error values are considered.

### 4.2. Statistical Metrics

#### 4.2.1. Statistical Measures of Eye Tracking Performance 

Gaze experiments are usually performed on a group of subjects with group sizes ranging between 5–15 or even 20–30 participants for improving test reliability. For analyzing gaze data from the numerous subjects, relevant statistical parameters on the collected data must be evaluated to draw significant inferences on collective data characteristics and insight into error patterns. 

***Method:***

The following statistical parameters were used to evaluate our eye tracking system:

$$\mathrm{Mean}\;\left(\varphi \right):\;1/n\overset{n}{\underset{i=1}{\sum }}x_{i},\;Z\;\mathrm{score}\;\left(\sigma \right):\;(x_{i}-\varphi )/\sigma ,95\%\;\mathrm{confidence}:\;\varphi \;\pm 1.96\sigma /\sqrt{n}$$

where σ is the standard deviation and *n* is the number of data points. The 95% confidence interval signifies the certainty that the mean error value would lie within the upper and lower bound of this interval. A larger confidence interval indicates higher variability in the data, whereas a small interval indicates more consistency of results. The Z score indicates the presence of unusual data points within the dataset, as gaze data quality may vary from person to person. It might be noted that typical research works in eye gaze rarely analyze their results using detailed statistical methods other than specifying the mean error values.

***Example Results:***

**Figure 5:**
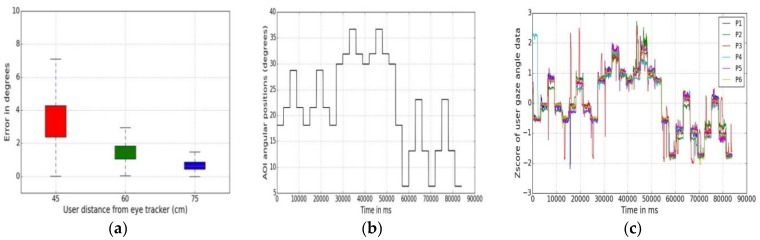
These plots demonstrate the use of statistical metrics to evaluate data from eye tracking experiment. (**a**) Shows a box plot of gaze data from three experiments UD45, UD60, UD75. (**b**) Shows the variation of ground truth angular positions of the AOIs during one experimental session done at 45 cm, as a function of time. (**c**) Shows the corresponding Z score variations of gaze angles for 5 users (P1–P5). The Z score represents how many standard deviations each data point is away from the mean and therefore shows the number of unusual points or outliers in the dataset. (**c**) Shows that level of gaze data scatter varies from person to person for same experimental conditions.

Examples of statistical analysis on gaze data are shown in Table 3 and Figure 5a–c. Data collected from user distances experiments (for 45 and 75 cm) are used for analysis. Implementing the metrics on gaze data reveals some important characteristics of the eye tracker under the impact of variable user distance conditions. It is seen that maximum gaze angle and mean gaze errors are higher when users are closer to the tracker (at 45 cm) than when they are further away (75 cm). Also, the confidence intervals are lower in UD75 than in UD45 experimental data, which means that error variability is more at lower user-tracker distances. Use of statistical metrics may therefore help to know how to get optimal good accuracy and better consistency of results from any given tracker.

A box plot is a valuable method to compare several error statistical attributes from multiple experimental datasets [44]. A box plot of the data from the three user distance experiments (45, 60, 75 cm) is shown in Figure 5a. It presents the statistical result summary, i.e., minimum, first quartile, median, third quartile, and maximum error values for the UD45, UD60 and UD75 experiments in a single figure. In each box the minimum and maximum data values represent the endpoints of the vertical line and height of the box shows interquartile range. A small interquartile range indicates that data is centered while a larger range means that the data is more scattered. The result of using box plot on UD45 and UD75 data indicates that UD45 dataset is more scattered in nature than UD75 data, and the error magnitudes are also higher. Another statistical metric, called Z scores analysis is done on UD45 gaze data to estimate the level of scatter in data points at different AOI positions and is shown in Figure 5c, which helps to reveal person-to-person variation of gaze data properties. 

#### 4.2.2. Histogram Based Metrics

To study the similarity between data from different eye trackers, experiments or comparison of data from a test experiment with a reference dataset, certain data similarity metrics based on histograms can be used. Such metrics for histogram comparison include correlation [45], intersection [46] and Bhattacharya distance [47]. 

***Method:***

If H_1_ and H_2_ are the histograms gaze errors from two datasets from the experiments, then d(H_1_, H_2_) is the histogram comparison metric and *N* is bin size. For the correlation and intersection measure, higher the result, the more accurate is the match whereas for Bhattacharya distances, lower the result, the better the match. The expressions for three different similarity measures for comparing two histograms are given below:

(11) $$\mathrm{Correlation}:\;d\left(H_{1},H_{2}\right)=\;\frac{\sum_{I}{(H}_{1}(I)\;-\;\bar{H_{1}}{)(H}_{2}(I)\;-\;\bar{H_{2}})\;}{\sqrt{\sum_{I}{(H}_{1}(I)\;-\;\bar{H_{1}})\;\sum_{I}{(H}_{2}(I)\;-\;\bar{H_{2}})\;}}\mathrm{where}\;\bar{H_{k}}=\frac{1}{N}\sum_{J}H_{K}\left(J\right)\;$$

(12) $$\mathrm{Intersection}:\;d\left(H_{1},H_{2}\right)\;=\sum_{I}\min\left(H_{1}\left(I\right),H_{2}\left(I\right)\right)\;$$

(13) $$\mathrm{Bhattacharya}\;\mathrm{distance}:\;d\left(H_{1},H_{2}\right)\;=\sqrt{1-\frac{1}{\sqrt{H_{1}H_{2}N^{2}}}\sum_{I}\left(H_{1}\left(I\right)\;-{\;H}_{2}\left(I\right)\right)\;}$$

***Example Results:***

Using these three metrics, the similarity measures between the gaze error data from a pair-wise choice of the user distance experiments (e.g., UD45-UD60, UD45-UD75, total 6 pairs) are calculated and presented below in Table 4. The values in each cell are the results obtained from using the above Equations (11)–(13) on a pair of histograms calculated on gaze error data from the experiments. It is observed that match between data from UD45 and UD75 experiments is low. Results from UD60 and UD75 experiments have better correspondence. This metric could be especially useful to compare data from multiple eye trackers captured under a wide range of operating conditions, in which it is difficult to assess data characteristics or correspondence by looking at just the error magnitudes.

### 4.3. Sensitivity Metrics

Gaze estimation systems operating in real life face several non-ideal conditions like changes in user head pose, user distance, variation in display size and resolution and if eye tracking is done on a mobile platform, then variation in platform orientation as well. Currently, there exist no quantitative metric to indicate how the accuracy of an eye tracker may be affected due to presence of each of these factors. Therefore, by running experiments on these factors (head pose, user distance, platform orientation variations, display characteristics) and using the collected data, several gaze-error-sensitivity metrics are derived, tested and presented in the subsections below.

#### 4.3.1. Head Pose Sensitivity

Head pose sensitivity analysis is done in this work to determine how the variations of a user’s head pose may affect the gaze tracking accuracy of a tracker under test. This is because the manufacturers or designers building the eye trackers do not quantitatively specify how much the tracking accuracy may deteriorate if user head pose is varied. For deriving this metric, data is used from our head-pose variability experiments as described in Section 3 above.

***Method:***

**Figure 6:**
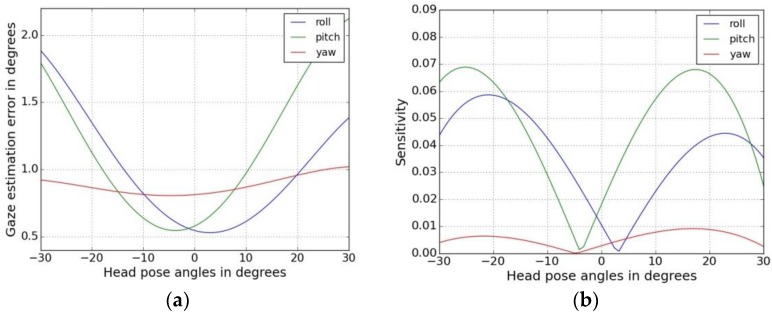
(**a**) Shows the variation of gaze estimation error as a function of head pose angles in roll, pitch, yaw directions. (**b**) Shows the variation of head pose sensitivity as a function of head pose. These plots help to know how much head movement is allowed to keep gaze error within limits.

The head pose sensitivity for head pose variation in roll, pitch, yaw (*r*, *p*, *y*) angles is defined as:

(14) $$\;S_{r}=\;\frac{\partial E_{r}}{\partial H_{r}},\;S_{p}=\;\frac{\partial E_{p}}{\partial H_{p}},\;S_{y}=\;\frac{\partial E_{y}}{\partial H_{y}}$$

where *S_r_*, *S_p_* and *S_y_* are the sensitivities with respect to head pose roll, pitch and yaw angles respectively. *H_r_*, *H_p_* and *H_y_* are the head pose angles in roll, pitch and yaw directions and *E_r_*, *E_p_* and *E_y_* are the corresponding gaze estimation errors (in degrees). The plot of gaze error vs. head pose angles is shown in Figure 6a and the head-pose sensitivity plots for head pose changes in roll, pitch and yaw directions are shown in Figure 6b.

***Example Results:***

From the head pose sensitivity experiments, it is seen that lowest errors occur for frontal head positions and sensitivity increases as magnitude of the head pose angles increases. Figure 6a also shows that the roll and pitch component of head motion affects gaze errors more strongly than yaw movements. Overall this metric helps to show that the head pose tolerance of the given eye tracker is quite low, and to achieve reliable gaze tracking, a user head movements must be constrained, and therefore the use of chin rest is essential.

#### 4.3.2. Platform Orientation Sensitivity

This is a new kind of study in which the impact of platform orientation on the accuracy of an eye tracker mounted on a tablet platform is observed. This is a vital analysis for eye tracking and gaze applications on handheld devices like smartphones and tablets, which face reliability issues due to the highly dynamic nature of these devices. However, the quantitative impact of device pose and its variation on eye tracking applications running on such platforms has not been explored yet.

***Method:***

**Figure 7:**
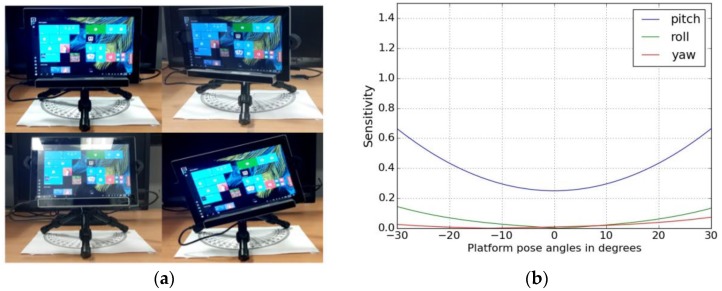
(**a**) Shows the different orientations of the tablet mounted with the eye tracker in neutral position (top left) and (clockwise from top) roll, pitch and yaw variations of the tablet+ tracker setup. (**b**) Shows the gaze error sensitivity to orientations of the tablet when eye tracking is performed on it.

For deriving this metric, the eye tracking experiment is run on a tablet mounted with the tracker and the platform orientation (in roll, pitch yaw angles) is altered. Some of the tablet pose variations (variation in roll, pitch and yaw angles with respect to the neutral frontal position of the tablet where roll, pitch, yaw = 0) for the eye tracking study are shown in Figure 7a. The tablet is mounted on a tripod along with the eye tracker and its orientations are changed. Then the same UI and workflow as shown in Figure 1 is used with participants to collect gaze data from the tracker and gaze errors are estimated for each platform position. The platform motion sensitivity metric is shown below:

(15) $$\;S_{pr}=\;\frac{\partial E_{pr}}{\partial P_{r}},\;S_{pp}=\;\frac{\partial E_{pp}}{\partial P_{p}},\;S_{py}=\;\frac{\partial E_{py}}{\partial P_{y}}$$

where *S_pr_*, *S_pp_* and *S_py_* are the platform orientation sensitivities with respect to platform orientation roll, pitch and yaw angles respectively. *P_r_*, *P_p_* and *P_y_* are the platform pose angles in roll, pitch and yaw directions and *E_pr_*, *E_pp_* and *E_py_* are the corresponding gaze estimation errors (in degrees). 

***Example Results:***

It is seen that for tablets, the gaze errors are most sensitive to pitch angle variations of the tablet + tracker pose (rotation about Y axis) while roll and yaw variations do not have appreciable effect. 

#### 4.3.3. Gaze Tracking Efficiency (Gaze Error Sensitivity to User Distance and Gaze Angle)

This metric is relevant to studying the impact of user distance and viewing angle on gaze error. Data from UD 45, UD60 and UD75 experiments are used to derive and test this metric.

***Method:***

**Figure 8:**
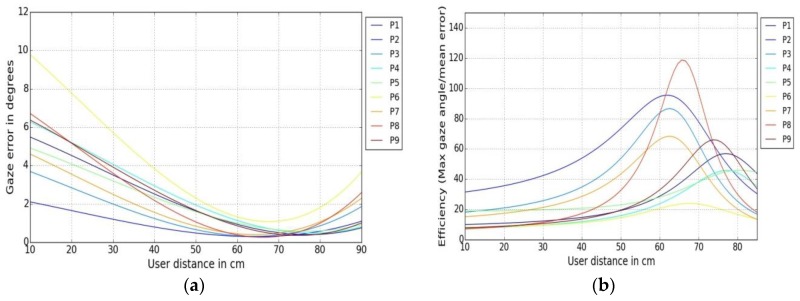
(**a**) On the left shows the variation of gaze estimation error (estimated using Equation (8)) with user-tracker distance for 9 users (Each line shows error data for one user). (**b**) Shows the gaze tracking efficiency varying with user distance from tracker, estimated using Equation (16). (**b**) Shows that for most users, the tracking efficiency reaches a peak at a distance of 65–75 cm from the tracker. This indicates the best operating distance for the tracker at which gaze errors are minimum.

Gaze data was collected from 9 participants (named P1 to P9) at 3 different user tracker distances (45, 60, 75 cm) for each person. The gaze estimation error vs. user gaze angle is plotted in Figure 8a below. An efficiency metric essentially gives an idea about how to obtain maximum output for given range of inputs. In this case, for a certain user distance, the gaze tracking efficiency measure is given by:

(Max gaze angle)_distance_/(mean error)_distance_ (16)

The gaze angles are estimated by Equation (5) above and interpolated for the user distances. The mean error here is the averaged gaze estimation error for a particular user for a certain user-tracker distance. Gaze efficiency for the different user distances is plotted in Figure 8b below.

***Example Results:***

In a remote gaze tracking setup, it is not known where the users should be ideally positioned in front of the tracker to achieve best results. The gaze tracking efficiency metric defined here may be useful to quantitatively estimate for which user distance and viewing angles the best tracking accuracy can be obtained for a given tracker for a range of user-tracker distances. Figure 8 shows that gaze tracking errors reduce (and efficiency increases) as the user distance from the eye tracker increases until a certain value, after which tracking errors show an increasing trend, with the best gaze tracking performance achieved between the distances 65–70 cm for the given tracker. This can be explained, as above a user-tracker distance of 70 cm, the errors increase (and tracking is ultimately lost around 80 cm) because user eyes are poorly detected by the eye tracker cameras. The possible reason for having worse errors at closer distances for the remote eye tracker could be explained by the fact that at shorter distances or larger viewing angles the eye tracker may not detect the eye pupil center accurately because the eye rotation angles are large. Similar results have been reported in [48,49]. However, for head-mounted eye trackers that have a scene camera, the gaze estimation errors may actually increase with user distance due to the influence of parallax errors which happens due to the spatial offset between the eye and the scene camera [50].

It may be noted that the exact dependency of gaze tracking errors on user distance for every tracker may be different depending on the tracker design or components (e.g., camera quality), and so this metric needs to be evaluated quantitatively for each tracker to obtain their best performance. 

#### 4.3.4. Error Spatial Density (Sensitivity to Spatial Locations of Targets on the Display)

Error magnitudes or statistics do not provide any idea about the actual spatial dependence of gaze error values on the screen where a user’s gaze is tracked. It cannot be understood if the error is uniform over the screen area or some parts of the screen are prone to more errors because of variability in the users’ visual acuity or due to display properties of the screen being used. For this purpose, a spatial error density metric is derived to represent the area wise distribution of gaze errors on the screen. 

***Method:***

The frontal display screen used during the experiments is divided into rectangular blocks around each AOI or stimulus target on screen. The error spatial density at each AOI is given by:

(17) $${\left(\frac{\sum_{i=1}^{m}\frac{E_{i\;}}{m}}{\left(µ\;*\;\mathrm{number}\;\mathrm{of}\;x\;\mathrm{pixels}\right)\;*\;\left(µ\;*\;\mathrm{number}\;\mathrm{of}\;y\;\mathrm{pixels}\right)}\right)}_{\mathrm{AOI}}$$

where, m is the number of gaze data points recorded by the tracker around each AOI (m ~ 90) while a user looks at them during an experiment session, E_i_ is the error at each data point estimated using Equation (8). The numerator therefore represents the mean error around each AOI (in degrees) and denominator is the total area of the rectangular block containing the AOI. 

***Example Results:***

This metric is further discussed along with visualization in Section 5, where the results from this metric are depicted as a heat-map of gaze error densities plotted around each AOI on the entire monitor screen area.

### 4.4. Gaze Error Analysis with Respect to Visual Eccentricity

The location of gaze targets on the display screen may have a significant effect on the quality of gaze data obtained from an eye tracker. Visual performance of human eye is improved (fast and accurate) when a stimulus target is presented more centrally to the fovea, and worsens when the target is further in the periphery of the retina. Accordingly, a given tracker may show good accuracy in lower visual eccentricity areas and worse performance in high eccentricity areas. Therefore, measuring gaze error characteristics with respect to stimulus eccentricity is valuable in specifying the level of gaze errors under various operating conditions for a given tracker. 

***Method:***

**Figure 9:**
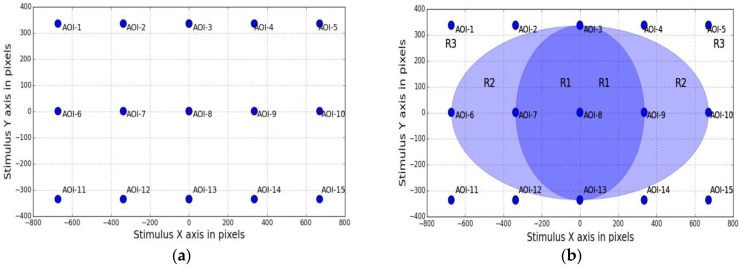

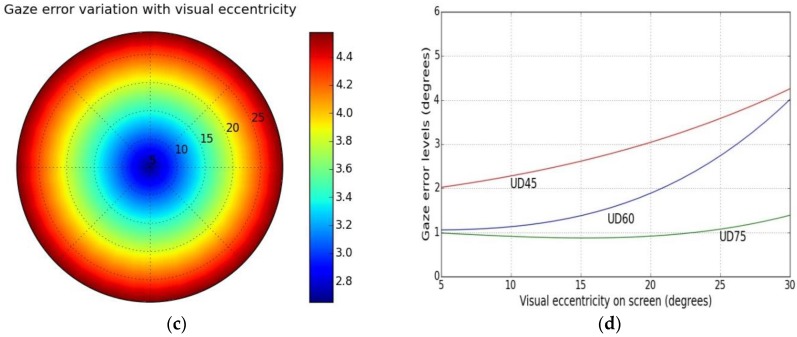
(**a**) The rectangular stimulus grid used in our experiments showing AOIs and AOI numbers. (**b**) Shows an eccentricity map constructed on the same stimulus grid, using data from UD75 experiments as an example. The dark blue inner ellipse region R1 has lowest visual eccentricity (11 degrees). The light blue larger ellipse region R2 has higher values (18 degrees) and the region outside R2 (region R3) has highest eccentricity (above 22 degrees). (**c**) This figure shows a polar plot of gaze error levels mapped with respect to visual eccentricity values (varying between 5 to 30 degrees) on the display screen, using data from UD45 experiment. It is seen that the gaze errors at the center are minimum and they increase to higher values with increasing visual eccentricities. The colorbar represents gaze error levels starting from low (blue) to high (red). (**d**) Shows the variation of gaze errors with visual eccentricity plotted using data from three user distances (45, 60, 75 cm). It is seen that when the user is close to the screen, gaze errors are more sensitive to visual eccentricities, whereas data for long user distances (e.g., UD 75) shows less sensitivity to eccentricities.

For this, our stimulus grid shown in Figure 2a was used to create an eccentricity map for the stimuli points and gaze error was analyzed separately for each eccentricity region. Figure 9a below shows our stimulus grid with its AOI (or stimuli) locations named AOI-1 to AOI-15. Figure 9b shows the rectangular stimulus grid superimposed with ellipses corresponding to the eccentricity of different stimuli locations. The parametric equation for ellipses centered around (0,0) may be stated as Equation (18) below, where a is the length of its semi-major axis and b is the length of its semi-minor axis and θ ranges from 0 to 2π:

x = a cos(θ), y = b sin(θ)(18)

In our case, the two elliptical regions R1 and R2 in Figure 9b were obtained by converting AOI locations of the rectangular grid of Figure 9a to respective polar coordinates using Equation (18). Coordinates of AOI numbers 3, 7, 9, 13 form the central (dark blue) ellipse which corresponds to lowest visual angles while AOI numbers 6, 10 fall in the higher visual angle regions of the light blue ellipse. AOIs outside the larger blue ellipse (e.g., AOI numbers 1, 5, 11, 15) are regions of maximum visual eccentricity. This way the rectangular stimuli area is split into different regions corresponding to different visual eccentricity values and gaze errors in each of these areas are separately analyzed. The magnitudes of visual eccentricities for each region are shown through the colormap of Figure 9b, where dark colors represent a lower visual angle and vice versa. For studying the impact of visual eccentricity on gaze errors, data from UD45, UD 60 and UD 75 experiments are taken and an example gaze error map for the eccentricity regions is presented in Figure 9c below. Table 5 shows the results comprising of visual angles corresponding to three eccentricity regions of the screen and corresponding gaze error values for 4 participants, using data from three experiments (UD45, 60, 75).

***Example Results and Discussion***

Table 5 above shows gaze error results from our UD45-75 experiments for four users, when the stimulus AOIs are split into different visual eccentricity regions. The table shows several significant gaze error characteristics when gaze data is analyzed with respect to visual eccentricity. Firstly it is seen that for all user distances, the Region 3 has the highest visual angles and also correspond to the high error levels for any participant. Therefore Region 3 which comprises of screen corners is not suitable for reliable gaze tracking for the given tracker, whereas Region 1 (or central regions of low visual angles) are more reliable. Secondly, it is seen that gaze errors at 75 cm distance are low and mostly similar for all eccentricity regions for all users. On the other hand, errors at 45 and 60 cm distance are strongly sensitive to stimulus eccentricity. This has the implication that at longer user-tracker distances (75 cm), the entire screen area may be used for reliable gaze tracking, whereas if a user is positioned closer (e.g., less than 60 cm), errors may increase sharply with stimulus eccentricities, and so the entire screen area may not be usable. For such close user distances eye tracking has to be limited within certain low eccentricity angles (e.g., within regions 1 or 2). 

The variation of gaze error with visual eccentricity is shown in Figure 9c above where gaze errors are interpolated and mapped to visual eccentricity values (starting with low values at the center to larger values outwards). This plot is created using UD45 data. Figure 9d shows the sensitivity of gaze error to visual eccentricity using data from three experiments (UD45, UD60, UD75). This plot signifies that for data collected above 60 cm, tracking accuracy is more uniform over the screen area, whereas for lower user distances, gaze errors are more sensitive to eccentricities. It may again be noted that these are example results and each tracker will have its own error characteristics with respect to eccentricities. Here the main aim is to present the eccentricity based tracker performance analysis procedure and demonstrate its significance using collected data.

### 4.5. The ROC Metric: Diagnostic or Subjective Performance Evaluation of Eye Tracking Systems

For in-depth performance analysis of an eye tracker using its data, the concept of a subjective performance evaluation metric rather than an objective one is introduced for the first time in this work. Objective or absolute performance measures like angular accuracy are independent of any prevalent criteria set by an observer while subjective performance measures may depend on specific accuracy thresholds set by the observer (observer is the person evaluating the system). With respect to eye trackers, this means that the absolute accuracy values may not be sufficient to know if a tracker is good or not, as a tracker may have different performance at different accuracy thresholds. 

For example, if a specific gaze application needs a minimum angular accuracy of 1 degree, a gaze tracker’s performance may be termed “bad” if there are a lot of data points where errors are higher than 1 degree. However, for the same tracker, if the minimum required accuracy by the application (or the accuracy threshold) is set to be 2 degrees, the performance may be called “good” if there are a very few number of points where errors are higher than 2 degrees. In other words, the tracker can track reliably if the accuracy threshold is set to 2 degrees, but will produce mostly errors if desired accuracy level is set at 1 degree. Thus performance is subjective to the set error threshold. However, there needs to be a method to know how a tracker would perform if these desired accuracy thresholds are set and varied between high and low. Such a method is described below.

***Method: The ROC (Receiver Operating Characteristic) Concept for Performance Evaluation***

To estimate a tracker’s performance with respect to different accuracy thresholds, the concept of ROC or Receiver Operating Characteristics [51,52] is implemented here using data from our gaze tracking experiments. Traditionally, ROC has been used in several cross-disciplinary fields like medicine [53] bio-informatics [54] and also computer vision for describing classification performance [55]. In this work, the ROC is used in determining the performance of our tracker when certain accuracy thresholds are set on its output data. This method proposed in this work is a new and experimental study and such an approach for evaluating eye tracking systems is different from any conventional analysis technique that has been done before. However, it opens up a novel way to look at performance characterization of eye trackers and helps to assess a tracker’s usability.

***Example Results: Implementation of Roc Concept for Subjective Evaluation***

To estimate a tracker’s performance with respect to different accuracy thresholds, the ROC concept is applied here. An ROC curve is plotted with the True Positive Rate (TPR) vs. False Positive Rate (FPR). In order to estimate the values of the TPR and FPR, one needs to estimate the number of true positives, true negatives, false positives and false negatives from the gaze tracker’s data. 

For estimating the true positives and negatives, we follow the analogy of estimating ROC for classifiers which need the Predicted values and Actual values. Starting with only raw error data for each gaze data point, we define the predicted values with respect to pre-set error threshold (ET) and actual values with respect to data mean M using the logic below:

The predicted value for a data point is positive if gaze error for that point > ET
The actual value for a data point is positive if gaze error for that point < M
The predicted value for a data point is negative if gaze error for that point < ET
The actual value for a data point is negative if gaze error for that point > M

The true positive (TP), true negative (TN), false positive (FP) and false negative (FN) values are therefore obtained as shown below in Table 6:

Then, for constructing an ROC curve, the TPR and FPR values are defined as:

(19) $$\;\mathrm{True}\;\mathrm{Positive}\;\mathrm{Rate}\;=\;\frac{\mathrm{TP}}{\mathrm{TP}\;+\;\mathrm{FN}},\;\mathrm{False}\;\mathrm{positive}\;\mathrm{rate}\;=\frac{\mathrm{FP}}{\mathrm{FP}\;+\;\mathrm{TN}}\;$$

To validate the proposed subjective performance evaluation principle, these equations are used to plot the ROC curves below for six different preset threshold error values (ET) of 0.5, 1.0, 2.0, 3.0, 4.0, 5.0 degrees on each of two gaze datasets from UD45 and UD75 experiments. The plots below show the effect of setting respective error thresholds (ET) to estimate TP, TN, FP, FN values and TPR and FPR estimates using the logic described above, and the corresponding changes in characteristics of ROC curves are clearly visible in the subsequent plots.

***Explanation and Analysis of Results***

**Figure 10:**
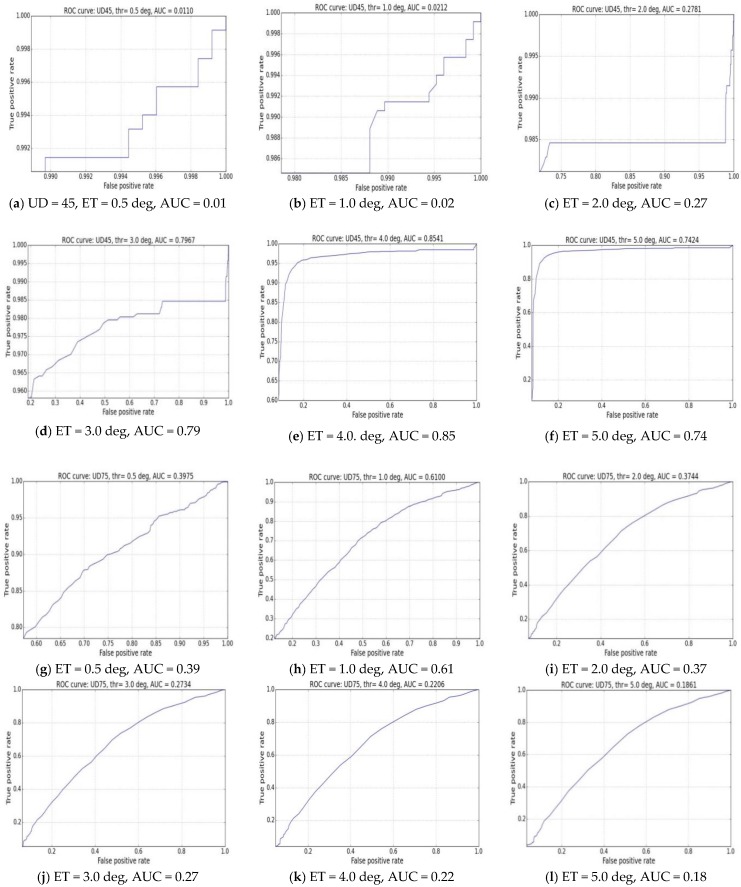
ROC curves for one user, setting different thresholds for gaze datasets (**a–f**) UD45 and (**g–l**) UD75.

Several error threshold values between 0.5 to 5 degrees are applied to construct the ROC curves. The area under the ROC curve (called AUC) is computed for all the plots. It is seen that for UD45 data, highest AUC values are obtained at error thresholds between 3 to 5 degrees. But the highest AUC values for UD75 data are found between threshold values of 0.5–1.0 degree. The changing AUC values for the two datasets, as seen from the Figure 10a–l indicate that for the UD45 data the probability of achieving best performance lies between 3 to 5 degrees of gaze tracking accuracy, whereas for UD75 data, best achievable accuracy lies between 0.5–1.0 degrees. The threshold intervals can be made even shorter (e.g., 0.5 degrees) to study corresponding variation of AUC values and determine best obtainable accuracy from the eye tracker. Similar results are observed for data from 13 participants. It may be noted that the data statistics computed on these two datasets (UD45 and UD75) and presented in Section 4.2.1 and Section 4.4 supports the ROC metric result. However, the benefit of the ROC technique is that users can set and observe the effect of varying error thresholds and determine how to obtain best performance from the tracker. They may also know if a certain tracker is suitable for them according to the required error threshold for a specific application. 

### 4.6. Discussion

This section introduced and defined several new gaze data metrics and analysis procedures that may reflect the all-round performance of a gaze estimation system by inspecting its data in great details to reveal patterns that are not visible by just looking at simple average accuracy measures. The methods described in this section use only the raw eye gaze data (x, y coordinates of tracked gaze locations) and ground truth (AOI locations on the UI) and therefore are independent of the tracking system or algorithm. This type of analytical methods may be useful to all kinds of gaze research and developmental works for complete evaluation of a gaze tracker’s system behavior and understanding of system capabilities and limits. The codes for the implementing the methods and metrics will be published in the project repository (https://github.com/anuradhakar49/GazeVisual) for use by the eye gaze research community in the coming months.

A few points regarding applicability of the metrics described above need to be discussed. In this work, the derivations of the gaze accuracy metrics have been done using data from both left and right eye. However, there may be eye tracking systems and gaze data collection software which provide only monocular data, i.e., data from one eye only. In these cases, the metrics will still work on the monocular gaze data, but the results might be different than that from using both eye data.

Another aspect is that there may be different tracker intrinsic parameters which affect eye tracking data quality and accuracy. These include choice of fixation detection settings, noise reduction and averaging window parameters to name a few. However, in this work we focused on studying the impact of extrinsic conditions on the tracker data characteristics, which arise mainly from changes in the operating environment of the tracker. The reason is firstly that impact of these operating conditions are sparsely investigated and quantitatively evaluated in eye gaze research. Secondly, there are commercial eye trackers which do not allow access to their device software or data parser settings and therefore there is no way to study the impact of intrinsic parameters on their data. Therefore, in order to propose a set of evaluation methods that can be applicable to any kind of eye tracker, the scope of this work is limited to studying tracker independent factors only.

Apart from defining quantitative metrics, the aspect of gaze error sensitivity to visual eccentricity is studied in Section 4.4, as it may have significant implications on the level of gaze accuracy obtainable from a tracker. A tracker may show low error levels at low visual eccentricities and high errors when eccentricities increase. The analysis method in Section 4.4 was therefore described which may be useful for eye tracking researchers as a basis for comparison of eye trackers as well as for determining tolerable eccentricities for a tracker under various operating conditions and also for designing proper stimuli for eye tracking applications. 

## 5. Visualizations for Evaluating Gaze Estimation Systems 

Graphical tools are key to viewing large volumes of gaze data and understanding data characteristics like error magnitude, bias and impact of variable operating conditions [29,56]. Researchers commonly design their individual test routines and report occurrence of errors mostly in statistical or tabular format. While software packages exist for gaze data analysis in some interdisciplinary areas like psychology, perception and usability research [57], there are no standard performance visualization/analysis tools available for researchers or developers working on gaze tracking algorithm/applications. Below, therefore some visualization methods are presented which may be used to study the accuracy of a generic gaze tracking system under variable operating conditions and estimate its overall performance. These visualization concepts are inspired from research fields like data science, visual analytics and statistics [58,59,60] and are implemented on data collected from our user distance, head-pose and platform movement experiments done using the test UI and workflow as shown in Figure 2a,b. The visualizations used in gaze research till now, as described in Section 2 are mostly focused on relating eye tracking data to human cognitive aspects (like attention, search patterns and interest) in the form of heat maps and fixation maps. However, dedicated visualizations for eye tracking performance analysis using raw gaze data from a tracker under test have not been developed in this much detail before. 

The visualizations presented in this work are divided into three subsections. The 1st subsection (A) comprises of methods for visualizing data from individual data files (e.g., files containing data of a single user or on a single experimental variable). The 2nd subsection (B) comprises of methods for aggregating and visualizing data (and its various properties) from multiple files (e.g., files containing data from multiple experiments or from multiple users or both). The 3rd subsection (C) presents the concept of the graphical software interface named GazeVisual which is being currently developed for easily accessing the gaze data metrics and visualization tools. As done in the previous Section 4 each visualization sub-subsection is split into “Method” describing the concept and “Results” describing the outcome and significance of the visuals tested on data from our different experiments.

### 5.1. Plots for Data Visualization from Single Eye Tracking Experiments

Subcategories in this include point and spatial error density maps, plotting of gaze angular field in the tracking space and 3D error magnitude plots. Special visualizations have been designed to represent impacts of head pose and platform orientation on gaze error levels.

#### 5.1.1. Data Density Maps 

Using data density maps, raw gaze data (gaze x, y coordinates collected at each target location of UI shown in Figure 1a) can be plotted as data point clusters, color-mapped according to point densities (calculated using histograms) around each AOI. 

***Method:***

**Figure 11:**
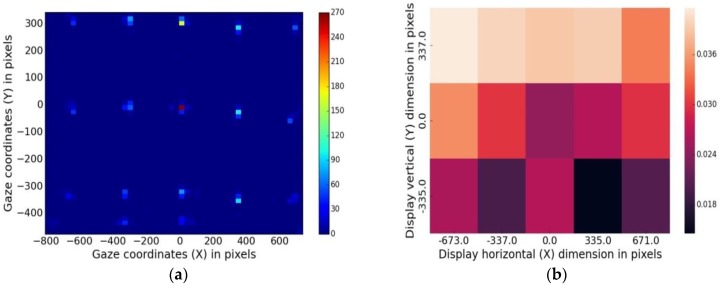
(**a**) Scatter density plot of gaze errors. (**b**) Shows a spatial error heat map for all AOI locations on the display screen (coordinate of screen center is 0,0) constructed from UD75 data. The gaze error density at all AOI locations has the units of degree/cm^2^.

Gaze errors are estimated using Equations (1)–(8) on data from UD45 experiment. This is then used to plot a color-mapped density plot from one participant and shown in Figure 11a. 

***Example Results:***

Figure 11a shows the gaze data point density around each target location on the overall screen area (data from UD45 experiment), with the scale alongside showing number of data points mapped to colors. This type of plots give a quick look into the relative scatter of data points at each AOI with a number scale alongside, which makes it easier to interpret the data patterns and detect any anomaly. 

#### 5.1.2. Spatial Error Heat Maps 

The concept of gaze spatial error density was discussed in Section 4.3.4, where the formula for gaze error density around each AOI on the display screen (Equation (17)) was derived. 

***Method:***

For visualizing the spatial distribution of errors on the display screen, a plot with gaze error densities around AOIs (using Equation (17) and data from UD60 experiment) color-mapped with values is shown in Figure 11b. The scale represents the color-map of error density values in degree/cm^2^. 

***Example Results:***

The gaze spatial error analysis heatmap can help to identify whether the gaze tracking accuracy is uniform over the monitor display and detect most probable locations for error on the screen. For example, from our study, the top left corners of the monitor were found to be prone to higher error values for all participants. This type of plots may help eye tracker users to improve gaze tracking performance, for example by checking the display screen quality, eye tracker mounting issues or compensating for the tracker’s performance at certain gaze angles if non-uniform or anomalous error densities are observed. It may be noted that error heat-maps are different from data density maps of Figure 11a, as the heat-maps take into account the display properties (screen size and resolution) of the computer screen where gaze is tracked. 

#### 5.1.3. Gaze Error vs. Visual Field

The visual field describes the extent of the observable area that is seen at any given moment. The UD45-75 experiments revealed dependence of gaze error values on user-tracker distances and user visual angles quantitatively, as discussed in Section 4.3.3. 

***Method:***

**Figure 12:**
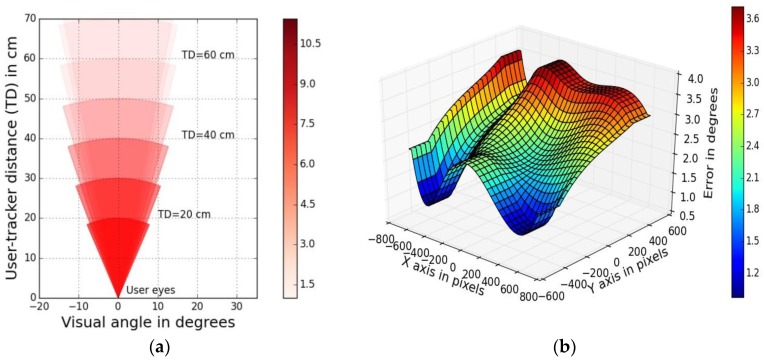
The sectors in the plot (**a**) on the left represent the visual angles of a user in degrees and are color-mapped according to gaze error obtained for each visual angle. The color-bar alongside shows the mapping to the magnitude of gaze errors in degrees. (**b**) Shows a 3D plot of gaze errors as a function of display X, Y dimensions (in pixels) for data from UD45 experiment along with error magnitudes mapped in a color bar alongside.

The variation of the visual angle with user distance from the tracker is shown as angular sectors in Figure 12a which also shows the dependence of gaze estimation accuracy on the user visual field. The tracker position with respect to the user is marked as tracker distance or “TD” in the plot. The magnitude of gaze estimation errors (derived from UD45-75 experiment data) for each visual angle is represented by the color intensity of the sectors. The corresponding color-map of gaze errors (in which low intensity of color means lower error) is shown alongside. 

***Example Results:***

This visualization of Figure 12a shows several interesting features, firstly the reduction in user viewing angle with increasing user distance from tracker (shown by narrowing sectors). Secondly, it shows that the given tracker achieves best accuracy at a narrow visual angle of less than 20 degrees (compared to normal human field of view which is about 60 degrees on either side of frontal eye position). It also shows that gaze errors increase significantly with even slight increase in user visual angles (demonstrating the tracker’s sensitivity to user distance). These kinds of plots, along with the visual eccentricity plots described in Section 4.4 (Figure 9c) can help eye tracking researchers to know where to position a user in front of an eye tracker for best tracking results. 

#### 5.1.4. 3D Gaze Error Distribution Plot

***Method:***

The 3D gaze error distribution plot shows the magnitude of gaze errors as a function of X and Y dimensions of the viewing area on the display screen. The gaze errors are plotted along Z axis and X and Y axis of the plot represent horizontal and vertical dimensions of the display in pixels, as shown in Figure 12b. Data from UD75 experiment for one user is used for the plot. The scale alongside shows the color-mapped magnitudes of gaze error values over the display screen area.

***Example Results:***

These plots help to diagnose gaze error levels over the display area. For example high error values are found to occur near the display corners and near the screen borders which could be due to high visual angles in those regions. Impact of display screen locations where gaze is tracked on corresponding gaze error levels has been reported in [61] which shows that gaze errors may increase by 33% on the screen border regions compared to those in the central regions.

#### 5.1.5. Platform Movement Tolerance Conics

A set of conics are used here to represent the degrees of freedom of movement “allowed” in a handheld device about its central axis if eye tracking is to be done on it with sufficient accuracy. The tablet angular pose variations in each of roll, pitch yaw directions, and corresponding gaze error values are derived from platform orientation sensitivity experiments as described in Section 4.3.2.

***Method:***

**Figure 13:**
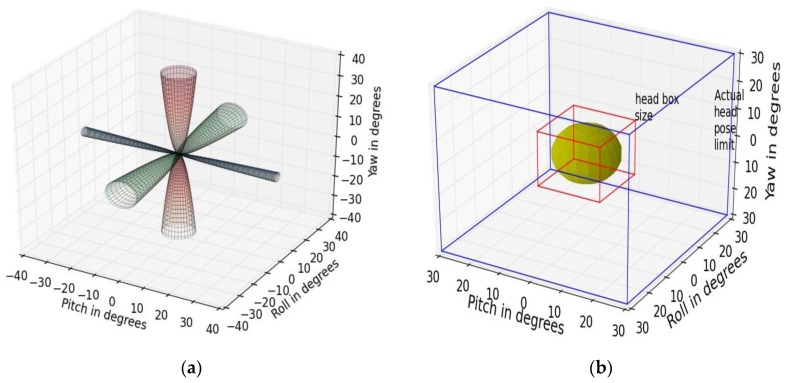
(**a**) Shows the freedom of movement of a dynamic platform like a tablet if one intends to perform eye tracking on it with sufficient accuracy. As seen from our experiments, gaze errors increase sharply with variations in the platform pose pitch angles and so the pitch movement of the tablet + eye tracker setup has to be kept within tight limits. As the impacts of yaw and roll variations are not very significant, larger pose variations is possible in those directions. (**b**) Shows the limits imposed by eye tracking on a desktop platform on head movement of a participant which has to be constrained within the small red box space to achieve reliable tracking accuracy.

Data from the platform orientation experiments are used to plot the conics in Figure 13a. The cones represent the range of platform angular pose variations (in roll, pitch, yaw directions), which occur due to variable hand poses when the tablet is held by a generic user. The aperture (or vertex angle) of each cone represents the maximum angle to which the edges of the device can be tilted with respect to the frontal position (where roll, pitch, yaw = 0) without gaze errors exceeding 1 degree. 

***Example Results:***

As observed from the platform orientation sensitivity curves in Figure 7b, the vertex angle of the cone corresponding to platform pitch movement is the smallest and about 5 degrees while the cone angle is about 10 degrees in the other directions of tablet roll and yaw. The utility of this visualization is that it shows the practical movement limits and maximum platform pose variations that are allowed for a certain tablet-tracker setup to perform reliable eye tracking on it. 

#### 5.1.6. Head-Pose Tolerance Box

***Method:***

This tolerance box visualization (Figure 13b) shows the degree of head pose variations allowed by the given eye tracker while maintaining acceptable accuracy levels and is implemented using data from our head-pose experiments as described in Section 2 and Section 4.3.1.

***Example Results:***

In the visualization of Figure 13b, the larger box shows the maximum degree of head movement (in roll-pitch-yaw angles) possible by an average user (whose head is shown by the yellow sphere) which is in the order of 30 degrees of angular movement in each direction of roll-pitch-yaw. The plot also shows that for reliable gaze estimation with the given tracker, the head pose of a user must be limited within the smaller “box”, the dimensions of which are about 10 degrees in each angular direction and correspond to gaze error values of 0.5 degrees. Such plots constructed for eye trackers may help to understand their head-movement tolerances quantitatively.

### 5.2. Plots for Data Aggregation from Multiple Eye Tracking Experiments

These plots are useful in clustering results from the multiple eye tracking experiments to derive inferences about gaze error patterns and dependence on various experimental variables, conditions etc. 

#### 5.2.1. Stacked Multi-Graphs

These kinds of plots could be used for displaying multiple parameters from several participants from one or more experiments on a single plot [62]. These reduce the need for creating a large number of separate plots and make it easier for a viewer to retrieve, understand and compare information obtained from multiple users. 

***Method:***

**Figure 14:**
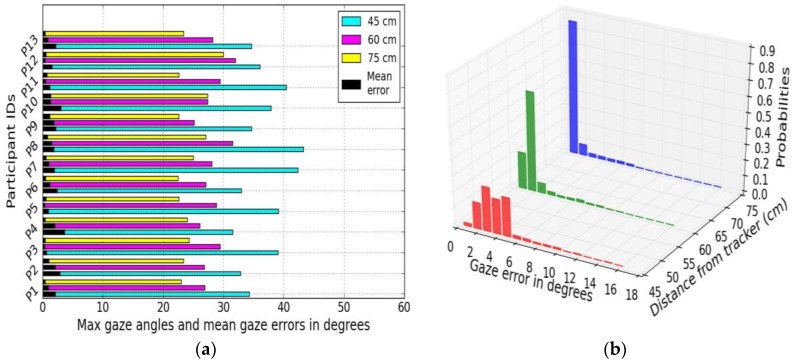
The figure on the left (**a**) shows gaze error and maximum gaze angle data from multiple experiments (UD45-75) for 13 participants (participant ID P1-P13) plotted together for easy visualization and comparison of results. (**b**) Shows gaze error data distributions from UD45-75 experiments plotted as 3D stacked histograms.

A stacked horizontal bar chart is constructed from our user distance experiments and shown in Figure 14a. In this, the mean gaze error and maximum gaze angles for three different distances for 13 different users (participant ID 1-13) are plotted together. The three colored bars represent three distances, the black bars’ length shows mean error levels for each user, and the length of the colored bars’ stands for the maximum gaze angle for each user.

***Example Results:***

This kind of plot helps to gain a quick look into a large gaze dataset obtained from any gaze experiment done with multiple users and draw inferences about the person-to-person variations of gaze data quality.

#### 5.2.2. Stacked Distributions 

For understanding data patterns, histograms provide more insight into data characteristics comparable to numerical values or statistics. 

***Method:***

Histograms are constructed using gaze error data from the experiments UD45, UD60 and UD75, and plotted together as stacked 3D histograms in Figure 14b. These histograms offer information on where error values are concentrated, presence of data extremes or unusual data patterns. 

***Example Results:***

The Figure 14b displays binned error values (number of bins = 20) for three different user distances and shows that with increasing distance, the errors move closer to lower error magnitudes. Stacked histograms plotted together on data collected from different experiments done under different conditions helps to see differences in gaze error patterns clearly and fast. These visuals could be useful to study impact of multiple experimental conditions on gaze errors from one person.

#### 5.2.3. Angular Error Charts for Multiple Experimental Variables

An angular chart can act as a powerful visualization method for plotting multivariate data while reporting performance of eye trackers [63]. When there is more than one factor, such as user distance, head pose angles that one needs to measure and compare while reporting tracker performance, this type of charts can come handy. In these charts, each variable under consideration is plotted on an axis that radiates out from a point (center value zero), with equal increments along each axis. These charts provide a compelling way of looking at data than simple tabular representations. 

***Method:***

**Figure 15:**
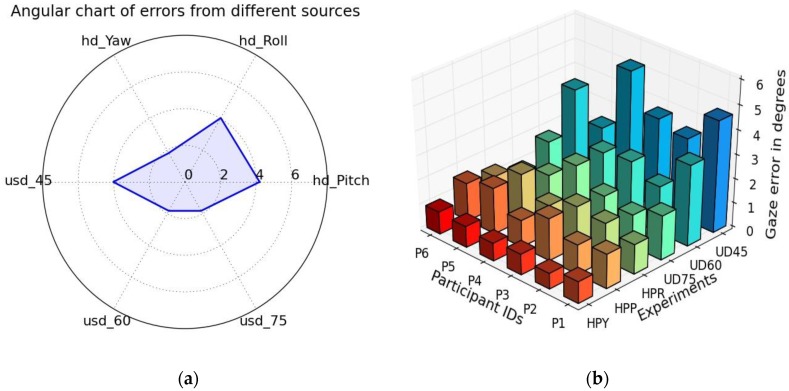
The figure (**a**) on the left shows the relative magnitudes of mean gaze error from different experiments for one person plotted on an angular chart. (**b**) On the right shows a very compact representation of data from all our experiments for 6 participants clustered into a single plot. HPY, HPP and HPR refer to head pose yaw pitch and roll experiments respectively.

Angular charts are implemented here to compare multiple variables from a number of our gaze experiments quantitatively, as shown in (Figure15a), and the aim is to make it easy to identify which experimental variables result in higher errors. 

***Example Results:***

This chart helps to understand the relative impact of factors like head movement, user distance etc. on the final gaze accuracy of the tracker. Each error source (e.g., each head pose direction or user distance) is treated as a “variable” plotted on an axis starting from the center (representing units of gaze angular accuracy) and all the variables are connected together to form a polygon [64]. All axes are equidistant and maintain same scale of angular accuracy on them. For example, from Figure 15a which aggregates data from our desktop based experiments, one can get the idea that the major error contributions in gaze error come from head roll and low user distances out of all other factors. 

#### 5.2.4. 3D Bar Clusters

***Method:***

This type of plot (Figure 15b) is implemented to aggregate data from multiple persons and all of our desktop based experiments to study and compare the gaze error levels of individuals across different experimental sessions. In the plot, different experiments are coded by colors with respective error values represented by bar heights, plotted along the Z axis. 

***Example Results:***

This kind of plot can be useful in inspecting data from a large number of experiments, done with multiple participants and judge overall eye tracking performance by gaze error levels which is shown along the Z axis. For example, the plot 15(b) helps to draw several meaningful inferences about our multiple experiments. It shows that minimum error levels occur from head pause yaw variation experiments and highest ones from UD45 experiments for majority of participants. Also it is seen that participant P2 has lower errors in all experiments compared to other participants. 

### 5.3. GazeVisual v1.1: Concept of a Performance Evaluation Tool for Eye Trackers

**Figure 16:**
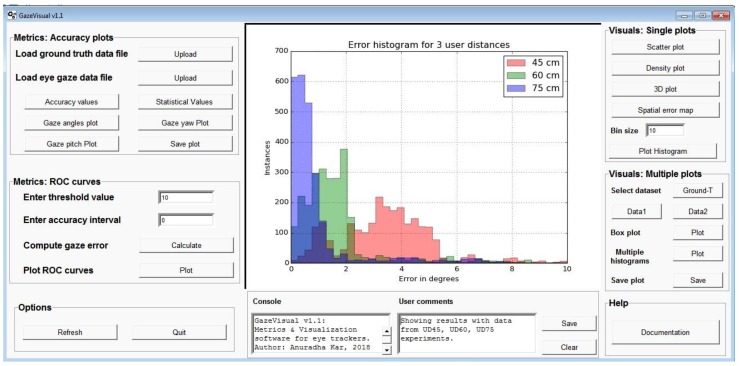
A snapshot of the graphical user interface GazeVisual v1.1 which is currently being developed for implementing the accuracy metrics and visualization concepts as discussed in Section 4 and Section 5 above. A sample histogram plot using data from UD45, UD60 and UD75 experiments is displayed in the plot area of the GUI. Other visualizations are also to be shown in this area and the plots can be saved. A demo video of the software demonstrating a set of its functionalities may be found in the GitHub link: https://github.com/anuradhakar49/GazeVisual/tree/Demo-videos.

This work is currently underway in which the metrics and visualizations presented in this paper are being packaged into a composite graphical user interface or GUI [65] format named GazeVisual v1.1, and a snapshot of its current look and operational features is shown in Figure 16 below. Using this GUI, a user will be able to browse and load the specific gaze data files along with ground truth data, compute and display results of various metrics (described in Section 4 and Section 5) by clicking on the buttons and see the visualizations in the plot window. 

### 5.4. Discussion

In this section, different methods for visualization of eye gaze data from several experiments with an eye tracker are presented. These visual analysis techniques would enable effective inspection of data characteristics of a gaze tracker and means for studying error pattern variations when any operating condition, such as user distance, screen properties, head movements is altered. Overall these visualizations are designed to aid in the complete characterization as well as comparison of multiple eye trackers. It may be noted that the only ingredient needed to implement these plots are gaze error magnitudes computed using Equations (1)–(8) from raw gaze and ground truth data and some experimental variables like user distance and display properties. This makes these visuals easily adaptable for any eye tracking system using its data samples and ground truth.

It may however be noted that this paper does not go deeper into the gaze data characteristics of the used eye tracker itself but only uses the collected gaze datasets to demonstrate the implementation of proposed visual techniques. The reasons for it are explained at the end of the Introduction section where we define the purpose and scope for our work.

The visualization concepts presented in this section are inspired from interdisciplinary areas like data mining, image processing and visual analytics. As per our knowledge, there hasn’t been any detailed work in gaze research so far for developing visualizations for studying data quality (especially to gain insight into gaze error levels, its distributions and spatial patterns) from generic eye trackers. The visualizations in this paper and the graphical software interface GazeVisual aids in this direction as also there is currently no publicly available software for performance evaluation of eye trackers. The implementation resources of the visualizations will be available in the open repository of the project (https://github.com/anuradhakar49/GazeVisual), for use and further development by the eye gaze community. The overall aim is to encourage eye gaze researchers to adopt more detailed ways of analyzing their eye tracker characteristics and providing open source tools and software for the same. 

## 6. Conclusions

There is a strong need for standard open source tools and common methods for measurement and data analysis in eye gaze research, without which each researcher has to develop their own methods or rely on expensive software which does not allow customizations. This leads to presence of inadequate details in gaze research results and increased diversity in reporting formats, making the interpretation of research results difficult and cross-validation of outcomes nearly impossible. There also needs to be an agreement in the metrics used for reporting gaze accuracy and standard methods for complete characterization of eye trackers under different operating conditions. 

Keeping these aspects in mind, in this paper, it is shown how the all-round performance of generic gaze tracking systems can be evaluated and compared numerically and visually. Several measures and visual methods for meaningful understanding of a gaze tracking system’s behavior, solely from its data outputs are presented. Some of these metrics are linked to visualizations while some are independent and vice versa, which shows that metrics and visuals are tightly related and both are essential for complete system description of an eye tracker. These methods could be useful for any researcher or developer of eye gaze systems to estimate and validate the performance of their system under widely variable influencing conditions. Especially, these evaluation tests may help to study the robustness of an eye tracker and degree of change in its data quality quantitatively, when operating under unpredictable and harsh conditions in unconstrained eye tracking applications. Also these can better describe the reliability of a newly designed system rather than error values expressed in simple numerical formats. Overall the presented metrics and visualizations are expected to help researchers as well as common users in understanding and improving the all-round performance of generic gaze trackers.

The GazeVisual v1.1 interface is an open source graphical tool which is being designed on the model of standard data visualization software but till date no such publicly available software for performance evaluation of gaze trackers exist. This interface would make the evaluation methods described in this paper extremely easy to use for a general user, who simply needs to load their gaze and ground truth data to the software to access all the evaluation metrics and visualizations through a few button clicks. 

The resources for implementing the concepts described in this paper will be released in an open repository where users can download these tools and modify them according to their own requirements and provide feedback for their improvement. The software components will be tested and accompany proper documentation to ensure seamless operation and easy adaptation by researchers. As future work in this direction, it is intended to include more system and external parameters into the evaluation criteria and study more user platforms for eye gaze applications such as eye trackers for large displays and head-mounted devices. 

## Figures and Tables

**Table 1:** Diversity in performance evaluation metrics used in eye gaze research articles.

Eye Tracking Platform	No. of Surveyed Papers	No of Papers with Metric: Degree	No. of Papers with Metric: Percentage	No. of Papers with Metric: Others (e.g., pixels, mm)
Desktop	69	44	16	9
Handheld	21	3	9	9
Automotive	35	11	14	10
Head-mounted	57	37	2	18

**Table 2:** Experimental setup details.

Eye Tracker and UI setup	Details	Display and Hardware Characteristics	Details	Experimental Variables
**Tracker type**	Desktop based, NIR LEDs + 1 Camera, 3 ps	**Screen Size**	Desktop: 22 inch Tablet: 10.1 inch	Single user, multiple user
**Calibration**	6 point	**Screen Resolution**	Desktop: 1680 × 1050 Tablet: 1920 × 1200	Fixed and variable user distance
**Tolerance**	Maximum user distance: 80 cm, spectacles allowed, chin-rest used	**Screen properties**	Desktop: 21.5 inch diagonal, width × height = 18.5” × 11.5” Tablet: 10.1 inch diagonal, width × height = 8.5” × 5.5”	Fixed and variable head pose
**User interface**	15 AOI locations, AOI radius: 10 pixels	**Pixel sizes of desktop and tablet screens**	Desktop: 0.275 mm Tablet: 0.113 mm	Screen resolution and pixel size
**Eye data type**	Fixation, AOI duration: 3 s, blinks allowed between AOIs	**Hardware details for desktop and tablet**	Desktop: Core i7, 3.6 GHz, 16 GB ram Tablet: Intel Atom X5, 1.44 GHz,4 GB ram	Platform orientation

**Table 3:** Gaze data error statistics for two user distances, data from same five users.

User Distance = 45 cm	Max Gaze Angle (degree)	Mean Error (degree)	95% Confidence Interval
User 1	34.24	3.63	3.54–3.71
User 2	32.83	3.96	3.88–4.04
User 3	39.04	3.42	3.35–3.49
User 4	32.99	4.61	4.51–4.70
User 5	34.69	3.52	3.44–3.59
**User Distance = 75 cm**	**Max Gaze Angle** **(degree)**	**Mean Error** **(degree)**	**95% Confidence** **Interval**
User 1	22.97	0.91	0.85–0.96
User 2	23.36	0.98	0.93–1.02
User 3	24.28	0.94	0.89–0.99
User 4	22.47	1.84	1.79–1.89
User 5	23.36	2.08	2.02–2.12

**Table 4:** Histogram comparison results between different user distance datasets.

Similarity Metric	Datasets Taken at User Distances 45, 60, 75 cm					
	45–45	45–60	45–75	60–60	60–75	75–75
Correlation	1.0	0.31327	0.18693	1.0	0.61210	1.0
Intersection	2457.07	1131.38	970.69	2502.46	1565.38	2514.01
Bhattacharyya	0.0	0.47526	0.52651	8.10 × 10^−10^	0.34091	8.10 × 10^−10^

**Table 5:** Gaze error analysis with respect to visual eccentricity, data from 4 users for 3 user distances. Units of visual angles in columns 3, 5, 7 are in degrees.

Eccentricity Region	User. Number	Visual Angles at 45 cm	Mean Error for 45 cm (degrees)	Visual Angles at 60 cm	Mean Error for 60 cm (degrees)	Visual Angles at 75 cm	Mean Error for 75 cm (degrees)
**R1**							
	User1	15.47	2.65	12.5	1.28	10.04	1.06
	User2	20.32	2.19	12.99	0.8	9.98	1.12
	User3	15.95	2.16	13.31	1.27	10.14	0.96
	User4	15.8	2.32	12.75	1.04	10.19	0.91
**R2**							
	User1	25.15	3.6	20.55	1.54	16.91	0.96
	User2	26.39	2.37	21.01	1.08	16.90	0.90
	User3	24.83	3.9	20.18	1.91	17.16	0.71
	User4	25.19	3.57	20.13	1.96	16.99	0.88
**R3**							
	User1	32.10	4.58	26.08	2.14	21.70	1.15
	User2	32.72	3.96	29.46	1.24	21.94	0.91
	User3	32.27	4.41	25.40	2.82	21.69	1.16
	User4	32.02	4.66	25.34	2.88	21.90	0.96

**Table 6:** Estimating the values of TP, TN, FP and FN using gaze error threshold.

Variables	Logic for Estimation
TP	Logical AND (Gaze error > ET, gaze error ≤ M)
FP	Logical AND (Gaze error > ET, gaze error > M)
TN	Logical AND (Gaze error < ET, gaze error > M)
FN	Logical AND (Gaze error < ET, gaze error ≤ M)
